# Stigma and discrimination against people living with HIV by health care providers in Egypt

**DOI:** 10.1186/s12913-023-09676-1

**Published:** 2023-06-20

**Authors:** Mirette M. Aziz, Shaimaa S. Abdelrheem, Heba M. Mohammed

**Affiliations:** 1 Department of Public Health & Community Medicine, Assiut University, Asyut, 71515 Arab Republic of Egypt; 2 Department of Public Health & Community Medicine, Aswan University, Aswan, Egypt; 3 Armed Forces College of Medicine, Cairo, Egypt

**Keywords:** HIV, Stigma, Discrimination, HPASS scale, Health care providers, Egypt

## Abstract

**Objective:**

HIV/AIDS has been recently increasingly observed in developing countries including Egypt. This study aimed to explore stigma and discrimination attitudes of health care providers (HCPs) in Egypt, as elimination of stigma in healthcare settings is a priority to improve case detection and management.

**Methods:**

A Google form questionnaire using the validated Arabic version of Health Care Provider HIV/AIDS Stigma Scale (HPASS) was sent to physicians and nurses of Ministry of health (MOH) hospitals and University hospitals in 10 randomly selected Governorates in Egypt. Data was collected from July to August, 2022 from 1577 physicians and 787 nurses. Bivariate and multivariable linear regression analyses were used to identify the predictors of the stigmatizing attitude of HCPs towards People living with HIV (PLHIV).

**Results:**

The majority of HCPs had worries about contracting HIV infection from their patients (75.8% of physicians and 77% of nurses). They believed that protective measures are not good enough to protect them from getting infected (73.9% of physicians and 74.7% of nurses). About half of the participants had worries about the safety of performing blood investigations to PLHIV (54% of physicians and 59.9% of nurses). Less than half of HCPs believed they have the right to refuse providing care to patients to protect themselves (44.6% of physicians and 50.1% of nurses). Only 10.5% of physicians and 11.9% of nurses have previously refused to provide health care to PLHIV. There was a significantly higher mean score of prejudice and stereotype among nurses compared to physicians (prejudice; 27.34 ± 7.88 vs 26.17 ± 7.5, stereotype; 18.54 ± 4.61 vs 16.43 ± 5.21, for nurses and physicians, respectively). Less years of physicians’ experience (B = -0.10, *p* < 0.01) and rural residence (B = 1.48, *p* < 0.05) were significantly associated with higher prejudice score while having lower qualification (B = -1.47, *p* < 0.001) was significantly associated with higher stereotype score.

**Conclusion:**

Standards of practice should be developed to adjust the services and prepare HCPs to provide medical care free from stigma and discrimination against PLHIV. Improving knowledge of HCPs regarding the methods of transmission of HIV, the use of infection control measures and the emotional factors shaping lives of PLHIV should be targeted through updated training programs. More concern should be directed to young providers in the training programs.

## Introduction

HIV/AIDS is one of the greatest challenges facing the world and has been recently increasingly observed in developing countries including Egypt [1]. Despite being a low HIV prevalence country, Egypt has witnesses a concentrated epidemic among people who inject drugs and men who have sex with men (MSM) in Cairo and Alexandria. In addition, Egypt is having a fast growing epidemic in the Middle East and North Africa Region (MENA) by a 76% increase in number of cases between 2010 and 2016 [2]. By the end of 2019, the total number of people living with HIV was estimated to be 22,000 in Egypt [3]. However, these numbers are definitely much less than the real numbers due to the obstacles hindering detection of all cases, of which stigma is a major one [4].

Despite the global effort to enhance HIV prevention, care and treatment, stigma against people living with HIV (PLHIV) in health care settings represent a major barrier to delivery and utilization of these services [5]. Stigma can discourage people from seeking HIV prevention information, testing, and treatment, due to fear of rejection from health services, thereby contributing to new infections and ultimately poor health outcomes [6, 7].

HIV-related stigma is defined as "a 'process of devaluation' of people either living with or associated with HIV and AIDS”. Discrimination follows stigma and is the unfair and unjust treatment of an individual based on his or her real or perceived HIV status" [6].

Stigma and discrimination are central issues in the fight against HIV because PLHIV often face social judgment and isolation with limited social interaction, violence and loss of employment [8]. Their identity and human rights are also negatively influenced by their HIV status [8, 9]. Moreover, there is a strong link between Stigma and discrimination by health care providers (HCPs) and the quality of the provided health care, which is critical for helping patients adhere to medication and maintain their overall health and wellbeing [10].

Studies from different parts of the world have revealed that there are three main causes of HIV-related stigma in health facilities, which are; lack of awareness among health workers about the consequences of stigma on PLHIV [11], fear of casual contact due to the incomplete knowledge about HIV transmission; and the association of HIV with immoral sexual behavior [5].

Stigma in healthcare settings has been recently addressed as a priority in Egypt in order to improve case detection and management and to progress towards achieving the third sustainable development goal which has a target to end the epidemic of HIV/AIDS by 2030 [12]. In collaboration between the UN Joint Team and the National AIDS Program, a national policy for stigma-free healthcare services was approved by Ministry of Health (MOH) and the protocol was disseminated to all health sectors. Moreover, partnerships with medical students have been invested, through student associations and Egypt’s Medical Syndicate, for disseminating key de-stigmatizing messages and relevant information, specifically during World AIDS Day and Zero Discrimination Day [3].

As we need to assess the impact of such programs and activities, we sought to explore the stigma and discrimination attitudes of health care providers in Egypt to compare the current situation with the previously detected high levels of discrimination towards PLHIV identified in the Egyptian DHS 2014, which revealed that stigma is almost universally associated with HIV/AIDS within Egyptian society, with only 2% of women having accepting attitudes towards PLHIV [13]. Updated data is critically needed for evidence-supported mobilization and allocation of resources to guide policy makers and program planners.

## Methods

### Study type

A cross sectional study.

#### Recruitment of study participants

Egypt is divided administratively into 27 governorates, four Urban Governorates (Cairo, Alexandria, Port Said, and Suez) and the other 23 governorates are subdivided into urban and rural areas. Nine of these governorates are located in the Nile Delta (Lower Egypt), nine are located in the Nile Valley (Upper Egypt), and the remaining five Frontier Governorates are located on the eastern and western boundaries of Egypt [13].

For sample recruitment, two urban governorates (Cairo and Alexandria), four governorates of Upper Egypt (Assiut, Aswan, Luxor and Qena) and four governorates of Lower Egypt (Sharkia, Dakahlia, Qaliobia and and Menofia) were randomly selected using the random digit generator. After numbering the governorates of Upper Egypt, Lower Egypt and Urban Governorates, a random sample was obtained from each category to cover the three types of Governorates, as each has distinctive cultural backgrounds which we hypothesized that it could affect HIV stigma attitudes. In each governorate, we sent a Google form questionnaire to physicians and nurses of the University Hospital and MOH Hospitals. The survey was announced through communication with the directorates of each governorate and the heads of departments of the hospitals included. It was distributed on Whatsapp groups and sent through e-mail. We tried to include the largest number of participants as possible through different means of social communication. We have extended the duration for response to allow as many physicians and nurses to participate. Physicians who were invited to participate were from the departments that may have direct contact with PLHIV. Departments such as clinical pathology, radiology and academic departments were not included in the study. Data collection was conducted from July to August, 2022. We extended the time through which participants can respond, as we know the busy schedule of physicians and nurses, to ensure the highest possible response rate. All questions in the “Google form” were designed to be obligatory answered, so we did not have incomplete questionnaires. All complete questionnaires were analyzed. About 5000 physicians and nurses were invited to participate in the study. Only 1577 physicians and 787 nursing staff completed the survey online, with a response rate of 47.3%.

#### The study questionnaire

Section 1 included questions on the study participants’ sociodemographic data, including age, gender, place of residence, marital status, professional qualifications, specialty, and years of experience.

Section 2 included assessment of HCPs attitudes towards PLHIV by the validated Arabic version of the HPASS scale. The validated Arabic version [14] consists of 18 items distributed through three subscales measuring prejudice (7 items), stereotypes (5 items), and discrimination (6 items), rated on a 6-point Likert scale ranging from 1 (strongly agree) to 6 (strongly disagree), with higher scores indicating more stigma towards PLHIV by HCPs. It has a Cronbach’s alpha of 0.91.

Section 3 included the practices of HCPs while dealing with PLHIV such as; avoiding physical contact with PLHIV, following special infection control measures, taking the patient’s consent before doing HIV test, taking infection control precautions with all patients whether their HIV infection status is known or not, refusing to provide health services to PLHIV if had the right to choose, and refusing to provide health care to PLHIV before. HCPs were also asked about the number of served HIV cases in the month preceding data collection. Some questions were obtained from a previous study [15].

Section 4 history of received trainings about infection control measures, preserving confidentiality of PLHIV, and stigma against PLHIV.

### Statistical analysis

Data were analyzed using SPSS Statistics for Windows (version 26.0). Descriptive analysis was performed for the participants’ socio-demographic data and participants’ responses to the likert scale of HPASS Arabic version and studied practices with PLHIV. The total score of each subscale was computed by adding the scores of the questions included in the subscale, and the mean score percent was calculated as score/optimal score of each scale × 100.

Independent Sample T test and Chi-square test were used to identify the significant factors associated with stigma of HCPs towards PLHIV. All significant variables on bivariate analysis were considered for inclusion in the multivariable linear regression analysis. *P* value < 0.05 was considered as significant.

## Results

Table 1 shows the characteristics of the study participants. The mean age of physicians was 33.63 ± 7.63 years and of nurses was 28.63 ± 6.6 years. There were slightly more males than females among physicians (55.8% and 44.2%, respectively), while more females than males among nursing (60.9% and 39.1%, respectively). The majority of participants were urban residents (89.3% and 74.1% among physicians and nursing respectively) and 58 % of physicians and 43.5% of nursing were married. About 62.0% from physicians and 57.7% from nursing staff were from Upper Egypt. Participants had variable degrees of professional qualifications, with the mean years of experience 8.59±7.47 among physicians and 6.14±6.40 among nurses.

**Table 1:** Characteristics of the study sample

Variable	Total (*n* = 2364)	Physicians (*n* = 1577)	Nurses (*n* = 787)
**Age**			
▪ Mean ± SD	31.96 ± 7.77	33.63 ± 7.63	28.63 ± 6.64
**Sex**			
▪ Male	1188 (50.3%)	880 (55.8%)	308 (39.1%)
▪ Female	1176 (49.7%)	697 (44.2%)	479 (60.9%)
**Residence**			
▪ Urban	1991 (84.2%)	1408 (89.3%)	583 (74.1%)
▪ Rural	373 (15.8%)	169 (10.7%)	204 (25.9%)
**Marital status**			
▪ Married	1250 (52.9%)	908 (57.6%)	342 (43.5%)
▪ Single	1114 (47.1%)	669 (42.4%)	445 (56. %5)
**Governorates**			
▪ Upper Egypt	1428 (60.4%)	974 (61.8%)	454 (57.7%)
▪ Lower Egypt	936 (39.6%)	603 (38.2%)	333 (42.3%)
**Professional qualification**			
▪ Bachelor’s degree	1006 (42.6%)	603 (38.2%)	403 (51.2%)
▪ Master’s degree	832 (35.2%)	782 (49.6%)	50 (6.4%)
▪ PhD	197 (8.3%)	192 (12.2%)	5 (0.6%)
▪ Nursing school	329 (13.9%)	–––-	329 (41.8%)
**Specialty**			
▪ Internal Medicine	1176 (49.7%)	722 (45.8%)	454 (57.7%)
▪ Surgery	930 (39.4%)	649 (41.1%)	281 (35.7%)
▪ Anesthesia and ICU	144 (6.1%)	110 (7.0%)	34 (4.3%)
▪ Dentistry	114 (4.8%)	96 (6.1%)	18 (2.3%)
**Years of experience**			
▪ Mean ± SD	7.688 ± 7.22	8.59 ± 7.47 6.00 (1–40)	6.14 ± 6.40 4.00 (1–36)

HCPs were asked about the number of served cases of HIV in the month preceding data collection. It was found the number of HIV cases ranged from 0 to 5 cases among physicians and from 0 to 4 cases among nurses. More cases were observed by physicians and nurses of medical specialties than surgical specialties.

Table 2 shows the attitude of health care providers towards PLHIV. It was observed that the nursing staff had more stigmatizing attitude towards PLHIV as compared to physicians. There were statistically significant higher mean total scores of prejudice and stereotype among nursing staff;27.34 ± 7.88 and 18.54 ± 4.61than among physicians; 26.17 ± 7.51 and 16.43 ± 5.21.

**Table 2:** Stigma of health care providers towards PLHIV

Variable ^a^	Total (*n* = 2364)	Physicians (*n* = 1577)	Nurses (*n* = 787)
**Prejudice**			
I worry about contracting HIV from HIV patients	1802 (76.2)	1196 (75.8)	606 (77.0)
HIV + patients make me uncomfortable*	1378 (58.3)	897 (56.9)	481 (61.1)
I would be hesitant to send HIV patients to perform blood investigations due to my fear of others’ safety	1292 (54.7)	852 (54.0)	440 (55.9)
It is a little scary to think I have touched HIV patients***	1330 (56.3)	828 (52.5)	502 (63.8)
I worry that general protective measures are not good enough to protect me from HIV patients	1753 (74.2)	1165 (73.9)	588 (74.7)
I would feel uncomfortable knowing one of my colleagues is HIV + **	1544 (65.3)	997 (63.2)	547 (69.5)
It would be hard to react calmly if a patient tells me he or she is HIV + ***	1209 (51.1)	764 (48.4)	445 (56.5)
Mean total score***	26.57 ± 7.65	26.17 ± 7.51	27.34 ± 7.88
Mean total score percent***	63.25 ± 18.23	62.32 ± 17.89	65.11 ± 18.77
**Stereotype**			
I believe most HIV patients got infected through risky behavior ***	1743 (73.7)	1115 (70.7)	628 (79.8)
I think HIV patients have engaged in risky activities despite knowing these risks**	1162 (49.2)	748 (47.4)	414 (52.6)
I think people would not get HIV if they didn’t have multiple sexual partners***	1067 (45.1%)	597 (37.9)	470 (59.7)
HIV + patients tend to have numerous sexual partners***	1113 (47.1)	685 (43.4)	428(54.4)
HIV + patients should accept responsibility for acquiring the virus***	1029 (43.5)	600 (38.0)	429 (54.5)
Mean total score ***	17.13 ± 5.12	16.43 ± 5.21	18.54 ± 4.61
Mean total score percent***	57.11 ± 17.1	54.77 ± 17.4	61.82 ± 15.4
**Discrimination**			
I believe I have the right to refuse to treat HIV patients for the safety of other patients *	822 (34.8)	523 (33.2)	299 (38.0)
I believe I have the right to refuse to treat HIV patients if other staff members are concerned about safety	1078 (45.6)	730 (46.3)	348 (44.2)
I would avoid conducting certain medical procedures on HIV patients	1123 (47.5)	734 (46.5)	389 (49.4)
I believe I have the right to refuse to treat HIV patients if I feel uncomfortable	1163 (49.2)	771 (48.9)	392 (49.8)
I believe I have the right to refuse to treat HIV patients to protect myself *	1098 (46.4)	704 (44.6)	394 (50.1)
I believe I have the right to refuse to treat HIV patients if I am concerned about legal liability	1341 (56.7)	893 (56.6)	448 (56.9)
***Mean total score***	19.62 ± 6.87	19.50 ± 6.84	19.84 ± 6.92
***Mean total score %***	54.51 ± 19.1	54.19 ± 19.0	55.13 ± 19.2

^a^HPASS questionnaire

The statistical tests were Independent Sample T test to compare difference in mean total score and total score percent between physicians and nurses. Chi square test was used to compare proportions between groups

^*^ *P* < 0.05, **P < 0.01, ***P < 0.001

Results show that HCPs had an evident prejudice attitude against PLHIV as the majority of them worried to contract HIV infection from their patients (75.8% of physicians and 77% of nurses) and that the protective measures were not good enough to protect them from getting infected (73.9% of physicians and 74.7% of nurses). They reported feeling uncomfortable knowing that a colleague is an HIV patient (63.2% of physicians and 69.5% of nurses) or having to deal with PLHIV (56.9% of physicians and 61.1% of nurses). Moreover, about half of the participants had worries about the safety of performing blood investigations to PLHIV (54% of physicians and 59.9% of nurses) or even touching them (52.5% of physicians and 63.8% of nurses). However, stereotype attitude was less observed as only about one third of physicians linked HIV infection to having multiple sexual partners and believed that patients should accept responsibility for getting infected.

It was found that just less than half of them believed they have the right to refuse providing care to patients if other staff members were concerned about safety (46.3% of physicians and 44.2% of nurses), to protect themselves (44.6% of physicians and 50.1% of nurses) or when feeling uncomfortable (48.9% of physicians and 49.8% of nurses). A considerable proportion would also refuse to conduct certain medical procedures for PLHIV.

**Fig. 1:**
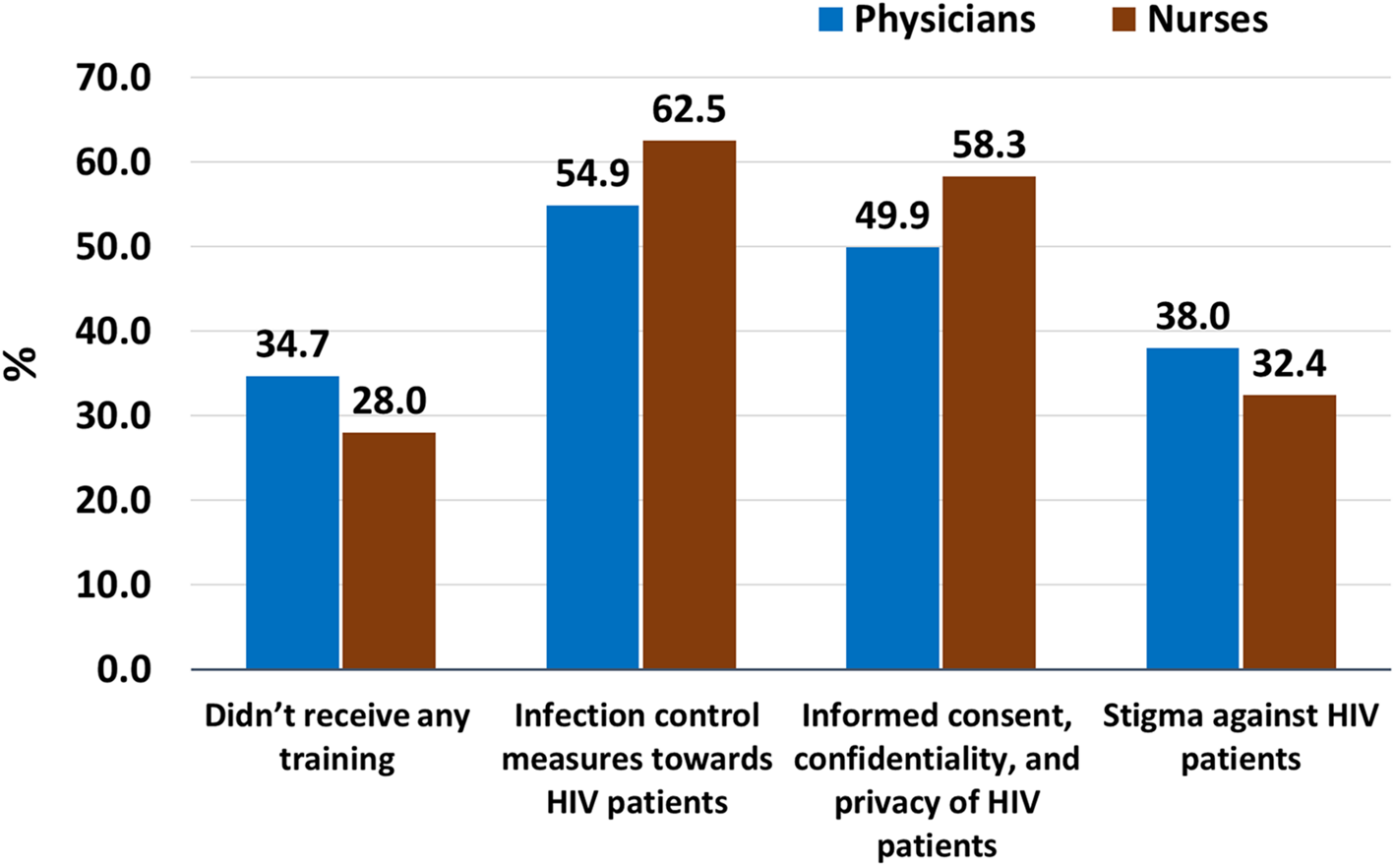
Training of HCPs about HIV related topics. *Multiple answers were allowed

Figure 1 shows that 34.7% of physicians and 28.0% of nurses didn’t receive any HIV related training. However, more than half of the participants (54.9% among physicians and 62.5% among nursing), received training about infection controls measures towards PLHIV, 49.9% of physicians and 58.3% of nursing received training about informed consent, confidentiality, and privacy of PLHIV. Only 38% of physicians and 32.4% of nurses received training about stigma against PLHIV.

Table 3 shows factors affecting the attitude of HCPs towards PLHIV. For physicians, less years of experience (B = -0.10, *p* < 0.01) and rural residence (B = 1.48, *p* < 0.05) were significantly associated with higher prejudice score, while being a male physicians (B = 1.27.*p* < 0.001) and having lower qualification (B = -1.47, *p* < 0.001) were significantly associated with higher stereotype score. Regarding discrimination**,** less year of experience (B = -0.10, *p* < 0.001), being married (B = 1.21, *p* < 0.01) and rural residence (B = 1.26, *p* < 0.05) were significantly associated with more stigma toward PLHIV among physicians. As for nurses, being married and having lower qualifications were the significant predictors of having more stigmatizing attitude towards PLHIV.

**Table 3:** Factors affecting stigma of HCPs towards PLHIV

Variables	Prejudice			Stereotype			Discrimination		
	**B**	**95% CI**		**B**	**95% CI**		**B**	**95% CI**	
		**Lower**	**Upper**		**Lower**	**Upper**		**Lower**	**Upper**
**Total**	***R*** ^**2**^ ** = 0.431**			***R*** ^**2**^ ** = 0.346**			***R*** ^**2**^ ** = 0.460**		
Years of experiences	-0.07**	**-0.11**	**-0.02**	0.01	-0.02	0.04	-0.07**	**-0.11**	**-0.02**
Males	0.12	-0.49	0.74	1.07***	**0.66**	**1.47**	0.32	-0.23	0.87
Married	1.18**	**0.50**	**1.86**	0.26	-0.19	0.70	1.43***	**0.82**	**2.04**
Rural residence	0.07	-0.16	1.54	0.49	-0.05	1.05	1.01*	**0.24**	**1.76**
Master’s degree and above	-1.53**	**-2.23**	**-0.82**	-2.27***	**-2.73**	**-1.80**	-0.44	-1.08	0.18
**Physicians**	***R*** ^**2**^ ** = 0.392**			***R*** ^**2**^ ** = 0.321**			***R*** ^**2**^ ** = 0.426**		
Years of experiences	-0.10**	**-0.15**	**-0.04**	0.003	-0.03	0.04	-0.10***	**-0.16**	**-0.05**
Males	0.18	-0.56	0.93	**1.27**	**0.75**	**1.78**	0.44	-0.23	1.12
Married	0.66	-0.15	1.49	0.28	-0.28	0.84	1.21**	**0.46**	**1.96**
Rural residence	1.48*	**0.29**	**2.67**	0.79	-0.02	1.61	1.26*	**0.17**	**2.34**
Master’s degree and above	-0.76	-1.65	0.12	-1.47***	**-2.09**	**-0.86**	0.08	-0.72	0.89
**Nurses**	***R*** ^**2**^ ** = 0.385**			***R*** ^**2**^ ** = 0.302**			***R*** ^**2**^ ** = 0.389**		
Years of experiences	-0.02	-0.12	0.06	-0.01	-0.06	0.04	-0.005	-0.08	0.07
Males	0.48	-0.67	1.65	1.07**	**0.38**	**1.75**	0.34	-0.68	1.36
Married	2.12**	**0.88**	**3.35**	0.24	-0.47	0.97	1.67**	**0.59**	**2.76**
Rural residence	-0.22	-1.48	1.02	-0.32	-1.06	0.40	0.74	-0.35	1.85
Master’s degree and above	-3.00**	**-5.18**	**-0.82**	-1.77**	**-3.04**	**-0.49**	-1.92*	**-3.83**	**-0. 71**

^*^ *P* < 0.05

^**^ *P* < 0.01

^***^ *P* < 0.001

Table 4 shows practices of HCPs in dealing with PLHIV. It was observed that physicians and nurses take specific infection control precautions when dealing with PLHIV (85.9% of physicians and 88.4% of nurses) and avoid physical contact with patients (76.6% of physicians and 84.8% of nurses), while only 30.1% of physicians and 34.9% of nurses follow the infection control measures with all patients regardless of their HIV infection status. It was also observed that only 64.0% of physicians and 76.2% of nurses take the patient’s consent before doing HIV testing. The unexpected good finding was that only 10.5% of physicians and 11.9% of nursing have previously refused to provide health care to PLHIV, despite the high observed rates of perceiving their right to refuse to provide care for PLHIV for different causes as previously shown in Table 2.

**Table 4:** Practices of HCPs on dealing with PLHIV

Variable	Total (*n* = 2364)	Physicians (*n* = 1577)	Nurses (*n* = 787)
Taking infection control precautions with all patients regardless their HIV infection status*	750 (31.7%)	475 (30.1%)	275 (34.9%)
On dealing with HIV patients:			
○ Avoid physical contact with HIV patients***	1875 (79.3%)	1208 (76.6%)	667 (84.8%)
○ Follow specific infection control precautions with HIV patients, not used for other patients	2050 (86.7%)	1354 (85.9%)	696 (88.4%)
○ Wear double gloves	1895 (80.2%)	1266 (80.3%)	629 (79.9%)
Taking the patient’s consent before doing HIV test***	1610 (68.1%)	1010 (64.0%)	600 (76.2%)
Refused to provide health care to HIV patients before	260 (11.0%)	166 (10.5%)	94 (11.9%)

^*^ *P* < 0.05

^**^ *P* < 0.01

^***^ *P* < 0.001

## Discussion

The study aimed to explore stigma and discrimination attitudes of HCPs in Egypt against PLHIV, as elimination of stigma in healthcare settings is a priority to improve case detection and management. We conducted a cross sectional study using an online Google form questionnaire. We used the validated Arabic version of HPASS. We recruited physicians and nurses from ten randomly selected Governorates all over Egypt.

Unfortunately, HCPs have been named as one of the most important sources of stigma against PLHIV. Several studies have documented HCPs discriminatory practices including patient neglect, provision of differential treatment based on HIV status, refusal of providing care, verbal abuse and breach of confidentiality [16, 17]. We believe that exploring stigma and its effects on HCPs behaviors and practices is vital for the development of effective stigma reduction interventions in Egypt, especially with the observed increased number of detected cases recently (Anecdotal evidence by communication with the sector of surveillance of infectious diseases, MOH, Egypt).

Up to our knowledge, there are only two tools for assessment of stigma of HCPs against PLHIV [14, 18]. This is the first study that used HPASS as a validated instrument in colloquial Arabic for assessment of stigma and discrimination of PLHIV by HCPs [14]. The scale was previously used in USA and Canada [19, 20]. These studies found that HIV continues to be a stigmatized disease, despite significant advances in care and concentrated efforts to reduce discrimination, stereotype and prejudice.

Our study found that prejudice and some aspects of stereotyping and discrimination against PLHIV prevail in this sample of Egyptian physicians and nurses. As previously documented in other studies [15, 21], the majority of physicians and nurses in our study believed that HIV cases should have got infected through risky behaviors (70.7% of physicians and 79.8% of nurses). However, they were less stereotyping regarding blaming HV cases of having multiple sexual partners (43.4% of physicians and 54.4% of nurses). A study in Tanta Governorate in Egypt found that 78.7% of HCPs reported that PLHIV should be ashamed of themselves because of their immoral behavior [15]. A qualitative study conducted on patients living with HIV/AIDS in Iran found that most patients perceived discrimination from health professionals and deprivation from medical treatment. Participants also described their experience of being labeled as prostitutes and facing sexual stigma in health care settings [21].

The majority of HCPs expressed their fears of contracting infection on dealing with HIV cases (75.8% and 77% of physicians and nurses, respectively). Their fears were evident as about half of the participants agreed on avoiding performing some medical procedures or testing for PLHIV and believing they have the right to refuse providing care to HIV cases to protect themselves. A study in Tanta Governorate in Egypt found that HCPs had also worries similar to those revealed in our study, as 21.3% felt worried to touch cloths of PLHIV, 26.4% were worried to dress the wounds of PLHIV and 27.4% were afraid to get blood sample from PLHIV [15].

A qualitative study in Rwanda showed that some HCPs held negative attitudes towards PLHIV as they believed they were sinners and that cultural and religious beliefs caused many HCPs to face some internal struggles when providing medical services to PLHIV [22]. In another study in Uganda, health providers reported that they “would feel very uncomfortable” handling MSM and the majority of them felt that they did not have adequate skills to effectively serve MSM [23]. Moreover, a study in Egypt found that 35, 48, and 43% of HCPs preferred not to provide medical services to injecting drug users, MSM and sex workers suspected to have HIV infection, respectively [15].

Participants in this sample have also followed specific precautions on dealing with infected cases by avoiding physical contacts (76.6% and 84.8% of physicians and nurses, respectively) and wearing double gloves (80.3% and 79.9% of physicians and nurses, respectively). Similar practices were observed in an earlier study in Egypt, but with much less frequencies than observed in our study (35.9% and 42.9% of physicians and nurses avoided physical contact and 42.9% and 52% of physicians and nurses wore double gloves) [15], denoting increased providers’ fears with time, rather than the expected effect of increased knowledge and assurance with increasing the awareness and educational programs directed against HIV stigma in the Egyptian community.

HCPs have also shown their worries of being not protected enough by the available protective measures. This finding coincides with the poor quality of infection control measures and scarcity of infection control supplies in developing countries in general [24, 25]. A previous study in Egypt has clarified these fears by performing in depth interviews with physicians and nurses. The study revealed the poor knowledge of HCPs about infection control measures against HIV infection and their disbelief regarding their efficacy. It has also expressed nurses concerns about the inevitable needle stick injury that cannot even be prevented by infection control measures [26]. In addition, a study in a South African tertiary hospital revealed that patients were sometimes tested for HIV without informed consents before surgery due to fear of being infected, thereby compromising patient’s confidentiality [27].

However, despite their fears, only 10.5% of physicians and 11.9% of nurses have refused to provide care to HIV cases when they were subjected to such situation, which reflects their ethical commitment to take care of cases and follow their institution laws and policies.

It is also worth mentioning that despite nurses were found to receive more HIV related training than physicians, they showed more stigmatizing attitudes than physicians and expressed higher frequencies of different worries. This could be attributed to performing most of the blood samples withdrawing tasks and cleaning linens and fomites of cases by nurses. However, this observation denotes the importance of targeting physicians in the training programs, who also might get in contact with patients’ blood in minor and major surgical procedures. Consistent with our results, higher discrimination and more worries among nurses were also observed in a previous study conducted in Egypt where nurses were more worried than physicians to take blood samples or measure temperature of PLHIV [15].

Such discriminatory attitudes can affect the service delivered by the HCPs and may deprive the patients from their health rights, which can be an important motive for people to hide their disease. This was clearly demonstrated in a previous survey in Egypt. The study showed that more than 40% of men and women did not reveal their HIV status while applying for health services, while more than half of those who revealed their HIV status were denied health services. Moreover, all women who revealed their HIV status to HCPs reported they have been denied sexual and reproductive health services. A small proportion was also found to be denied health insurance and forced to submit to a medical or a health procedure [28].

In this study, years of experience and higher qualification were negatively associated with stigmatizing PLHIV. This could be attributed to improved knowledge about HIV and its mode of transmission among older more experienced physicians and nurses or to the more profession in using infection control measure with higher qualifications. This finding was supported by another study [29].

We have also found that rural residence of physicians was associated with negative attitudes towards PLHIV, as rural communities in Egypt are very conservative with prevailing cultural factors that prohibit and stigmatize out of marriage relations and having multiple sexual partners, which have been linked to being infected by HIV infection, which denotes that sociocultural values and norms strongly influence providers attitudes, even in the presence of high level of medical knowledge. The strong association between sociocultural factors and stigma against PLHIV have been also previously documented in several studies [17, 30].

## Strengths and limitations

The study is the first study to address HCPs stigma against PLHIV using the validated Arabic version of the standardized tool of HPASS. It included a large number of physicians and nurses from all over Egypt; Urban, Upper Egypt and Lower Egypt Governorates. However, being based on self-report of stigma may underrepresent the views of the participants. An observational qualitative study may have enriched the findings of the study.

## Implications

As Egypt is one of the countries facing a growing HIV epidemic, the need for enhanced HIV medical attention and services is on the rise. In order to improve case detection, retain PLHIV on therapy and to manage their medical problems, the health care system needs to adjust the services and prepare HCPs to provide medical care free from stigma and discrimination in different health settings. We recommend the development of standards of practice for HCPs working with PLHIV and improving their knowledge regarding the methods of transmission of HIV and the use of infection control measures. Providers’ training should also include the social and emotional factors shaping lives of PLHIV. Educational programs to improve the whole community knowledge about HIV, correct misinformation, and eliminate HIV directed stigma are required to reduce the effect of entrenched cultural beliefs against PLHIV. Regular monitoring and reporting systems on stigma and discrimination in health care settings should be established to monitor the progress made in addressing HIV-related stigma.

## Data Availability

The datasets used and/or analysed during the current study available from the corresponding author on reasonable request.
